# Melanism, body size, and sex ratio in snakes—new data on the grass snake (*Natrix natrix*) and synthesis

**DOI:** 10.1007/s00114-020-01678-x

**Published:** 2020-05-12

**Authors:** Stanisław Bury, Tomasz D. Mazgajski, Bartłomiej Najbar, Bartłomiej Zając, Katarzyna Kurek

**Affiliations:** 1 Institute of Environmental Sciences, Jagiellonian University, Gronostajowa 7, 30-387 Kraków, Poland; 2 NATRIX Herpetological Association, Legnicka 65, 54-206 Wrocław, Poland; 3 Museum and Institute of Zoology, Polish Academy of Sciences, Wilcza 64, 00-679 Warsaw, Poland; 4 Faculty of Biological Sciences, University of Zielona Góra, prof. Z. Szafrana 1, 65-516 Zielona Góra, Poland; 5 Institute of Nature Conservation, Polish Academy of Sciences, Mickiewicza 33, 31-120 Kraków, Poland

**Keywords:** Ectotherm, Melanism, Polymorphism, Body size, Reproductive mode, Reptile

## Abstract

It is postulated that melanism in ectotherms is adaptive by enhancing thermoregulation, subsequent resource acquisition, and growth. Such effects may differ between the sexes as a result of the differential costs of self-maintenance and reproduction, but empirical support for the sex-specific consequences of melanism remains inconsistent. We studied the effects of melanism on body size and sex ratio in a population of the European grass snake (*Natrix natrix*) in SE Poland and also carried out a systematic review of the literature on the consequences of melanism in terrestrial snakes. Melanistic grass snakes of both sexes appeared to be smaller than the typical phenotype, which indicates higher predation pressure and minimal thermal benefits for black individuals. A female-biased sex ratio was observed in the typical phenotype, but not in melanistic snakes, suggesting that the costs for females and/or benefits for males are higher in melanistic individuals. In conjunction with earlier studies, our data indicate that the consequences of melanism may be related to the reproductive mode of species. In viviparous species, melanism tends to improve growth and/or body size and is more frequent in females, whereas the opposite holds for oviparous snakes. Further studies on melanism should examine a wider array of species with different reproductive strategies and traits beyond the usual thermal benefits.

## Introduction

Body coloration is a multifunctional trait often characterized by sophisticated variation (Kemp et al. 2005). Therefore, discontinuous phenotypes are generally thought to bear fitness costs as a result of the primary functions of a given color variation having been lost. The maintenance of such phenotypes within populations, i.e., color polymorphism, thus represents an interesting evolutionary phenomenon (Forsman 1995a; Forsman et al. 2008).

Melanism is an example of color polymorphism in which a phenotype is characterized by an overconcentration of melanin compared with the typical color morph (Trullas et al. 2007). In small vertebrates, melanistic individuals are known to bear an elevated risk of predation (Andren and Nilson 1981). However, there are positive consequences of a melanistic phenotype, including a better capacity to cope with parasites (Roulin et al. 2001), an improved anti-UV protection (Dubey and Roulin 2014), and a higher concentration of sex hormones (Ducrest et al. 2008) which prevents its complete removal from the population. The occurrence of melanism in ectotherms is particularly interesting, owing to its predicted positive effect on thermoregulation, including increased heating rate and higher achievable body temperature (Forsman 1995b; Trullas et al. 2007). Such thermoregulatory consequences are predicted to have a positive impact on the life-history traits of black individuals, including their body size—a prerequisite of survival and fecundity (Olsson 1993; Civantos et al. 1999). The improved thermoregulation anticipated for the melanistic phenotype is also likely to have sex-specific consequences, because of the sex-specific variation in the costs of reproduction and self-maintenance (e.g., Madsen and Shine 1993). In other words, the sex that bears the highest costs should benefit more from being black-colored, and, consequently, should grow larger and/or include a higher proportion of black individuals or be more frequent in the melanistic fraction (Forsman and Ås 1987; Luiselli 1992).

Snakes are frequently studied in the context of the consequences of melanism because this phenotype is common in many snake species. However, available data do not seem to provide a consistent picture of the sex specificity of the effects of melanism. A number of studies of viviparous species, including European vipers (*Vipera berus* and *Vipera aspis*), show a tendency towards greater size in melanistic individuals of both sexes and an elevated proportion of black females, which are the sex that bears the highest costs of reproduction (e.g., Monney et al. 1995, 1996; Madsen and Stille 1988). Available data on oviparous species are less conclusive, but the opposite effects of melanism have been observed concerning both body size and sex-specific phenotype proportion or sex ratio (e.g., Luiselli 1995; Zuffi 2008). Further research is needed to understand and clarify the direction of the effects of melanism on body size and sex ratio in these species.

Our aim in this study was to investigate the association of melanistic phenotype with body size and sex structure in a widespread oviparous reptile, the European grass snake (*Natrix natrix*). In this species, there is a pronounced sexual size dimorphism, i.e., females are larger than males, and the numbers of each sex in a population are roughly equal (sex ratio 1:1) (Borczyk 2007). The occurrence of a melanistic phenotype, though widely documented in this species (e.g., Nilson and Andren 1981; Böhme and Wiedl 1994), appears to be discontinuous. Moreover, little is known about the effects of melanism on body size and sex ratio. Although increased predation pressure has been confirmed in melanistic grass snakes (Madsen 1987), the widespread occurrence and persistence of the black phenotype indicate a beneficial side. In line with previous studies, we predict that the melanistic phenotype will achieve a larger size (Madsen and Stille 1988). With regard to the sex ratio, we expect females to be the sex that benefits most from being black-colored and to outnumber males in the melanistic fraction of the population. This is because male combat behavior is negligible in the grass snake (Borczyk 2004), so the costs of reproduction are clearly lower for males than for females. In addition, we reviewed the published data on sex-specific correlates of melanism in terrestrial snakes to find out whether any general patterns could be discerned. For this, we took the reproductive mode into account, owing to the fundamentally different costs of reproduction between viviparous and oviparous species.

## Methods

The study was conducted in the Bieszczady Mountains (SE Poland; 49° 14′ 5.28″ N 22° 33′ 30.91″ E). The presence of the melanistic phenotype in grass snakes in this area was described back in the 1970s and repeatedly confirmed in subsequent reports (Błażuk 2007). Here, the snakes were surveyed monthly from April to September and searched for in a wide range of different habitats, including but not restricted to forests, meadows, riverbanks, ecotonal zones, and anthropogenic sites. Individual grass snakes were identified based on external features, such as ventral color pattern and/or scale clipping. For each snake captured in the study, we determined the sex and measured the snout-vent length (SVL). Only data from sub-adult and adult snakes for which sex could be reliably identified were considered in this study (≥ ~ 30 cm SVL; Bury and Zając 2019). Fieldwork was carried out between 1981 and 2013. Before 2009, measurements were made only on melanistic snakes, but in subsequent years included individuals of both phenotypes. Altogether, our data included 103 specimens, out of which 67 were melanistic. None of the snakes was ever recaptured, probably because of the large extent of the study area.

To get insight into the possible effect of color phenotype and sex on SVL, we analyzed SVL data using a general mixed model, with phenotype, sex, and interaction of both as fixed factors; the source of the data was a random factor. To account for the possible effect of the data source (i.e., the period of sampling—before and after 2009), we included it as a random factor in the analysis. To explore the possible association between phenotype and sex ratio, the sex ratio within each phenotype (i.e., melanistic and typical color morph) was analyzed using the chi(Andren and Nilson 1981) test. All the analyses were performed in the Statistica software (version 13.1; StatSoft Poland).

We carried out a systematic review of the literature by scanning the Web of Science, Scopus, and Google Scholar using the keywords: “snake,” “reptile,” “melanism,” and “coloration”. We extracted papers that reported on the association of melanism with at least one of the following variables: body size, mass, condition, growth, sex ratio, and sex-specific melanism prevalence. For each species, we also noted the reproductive mode based on the reptile database (http://www.reptile-database.org/).

## Results

**Fig. 1:**
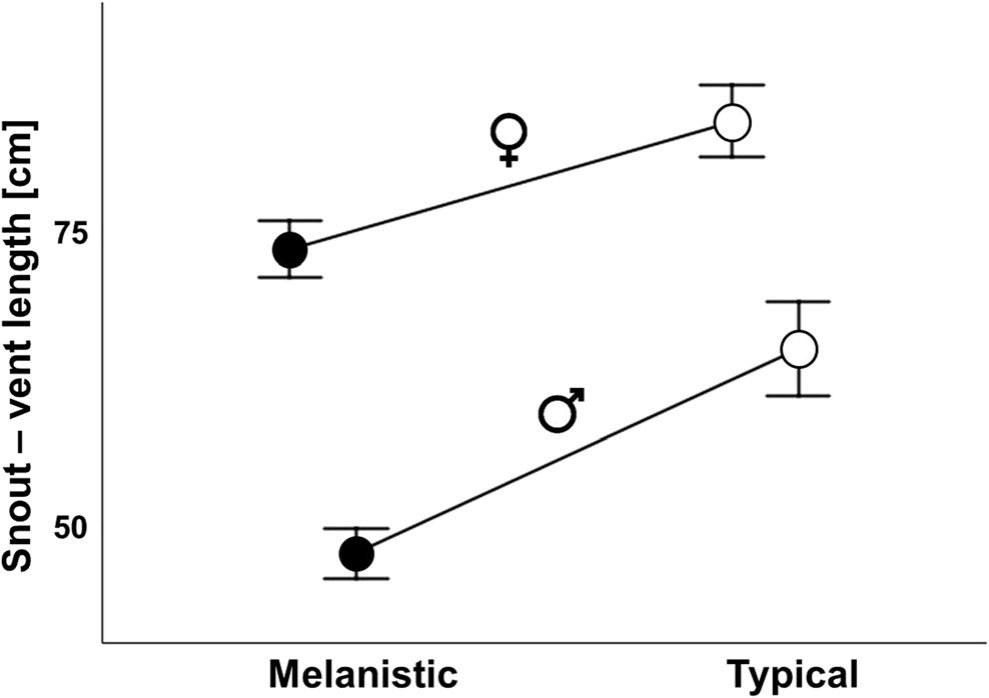
The impact of phenotype on sex-specific body size (SVL – snout-vent length) in the grass snake (*Natrix natrix*) (least square means ± standard error). There are significant differences between the sexes in both phenotypes (*p* < 0.001), as well as between both phenotypes (*p* < 0.001). Interaction term was non-significant (*p* = 0.24)

The general mixed model revealed significant effects of phenotype (F_1,98_ = 16.61; *p* < 0.001) and sex (F_1,98_ = 68.02; *p* < 0.001) on SVL, whereas the interaction of both factors appeared to be non-significant (F_1,98_ = 1.38; *p* = 0.24). The random effect of data source was not significant (F_1,98_ = 0.578; *p* = 0.45). Females appeared to be larger than males by ca 30% in the typical phenotype and 56% in the melanistic one (Fig. 1). Melanistic males were ca 29% smaller than typical ones, whereas melanistic females were ca 14% smaller than typically colored ones (Fig. 1). The descriptive statistics based on the raw data (mean ± SD; min-max; sample sizes, and sex ratio) are summarized in Table 1.

**Table 1:** SVL (snout-vent length; in cm) of two color morphs of grass snakes (*Natrix natrix*) (mean, standard deviation, range in brackets) in relation to sex. The sample sizes (*N*) are given

	Males mean ± SD (min–max) *N*	Females mean ± SD (min–max) *N*
Typical	66.49 ± 12.4 [cm] (43–84) *N* = 12	86.13 ± 13.06 [cm] (44.9–104) *N* = 24
Melanistic	47.14 ± 9.57 [cm] 27–73 *N* = 39	73.66 ± 16.19 [cm] 39.1–98 *N* = 28

**Fig. 2:**
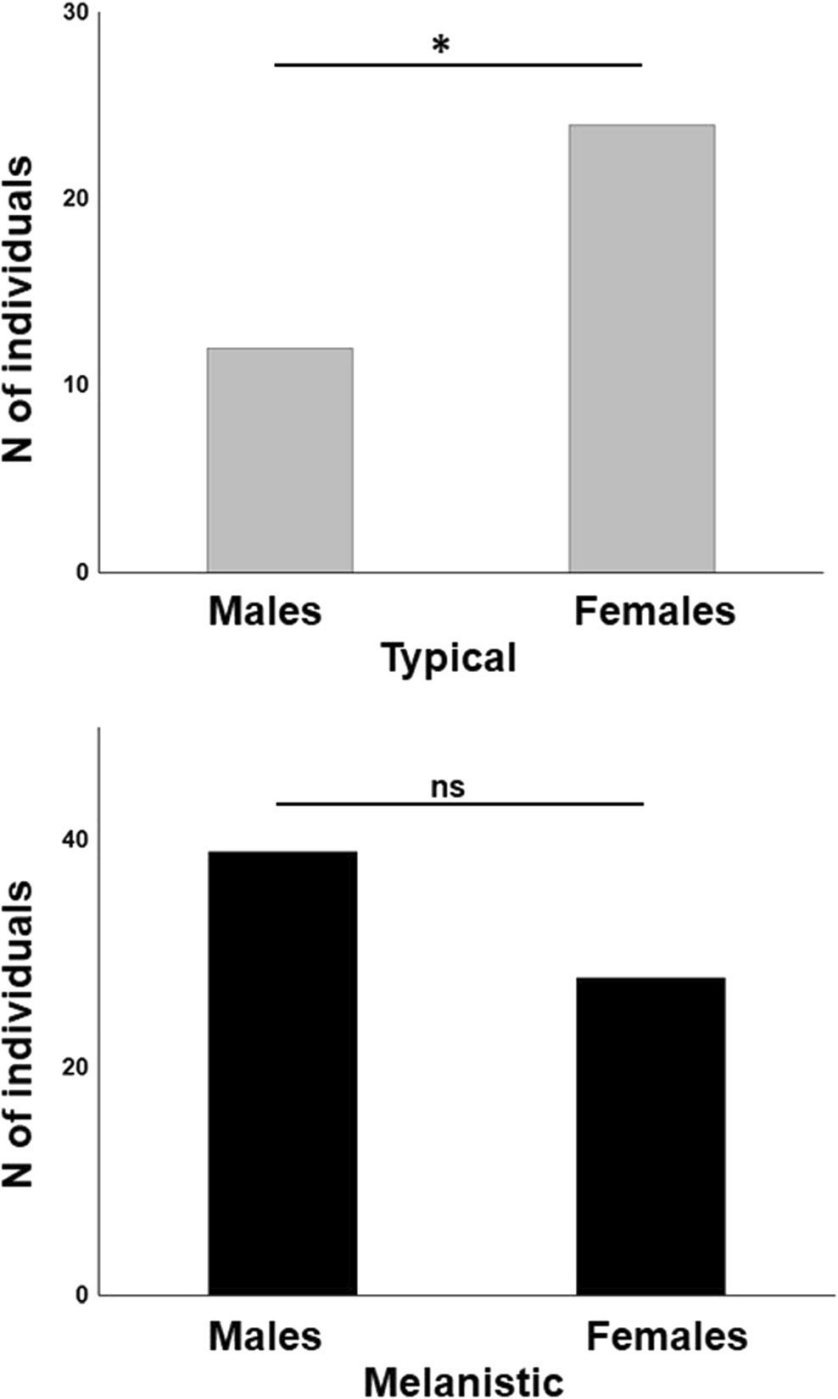
Sex ratio in both phenotypes of the grass snake (*Natrix natrix*) expressed as the number (*N*) of individuals representing each sex within each color phenotype. Females of the typical phenotype significantly outnumber males (ratio 1:2; *p* = 0.04), whereas no such effect is observed in melanistic snakes (ns – non significant; ratio 1:0.72; *p* = 0.18)

The sex ratio in the typical phenotype deviated from 1:1 towards a higher proportion of females (chi(Andren and Nilson 1981)=4.0; *p* = 0.04; Fig. 2), whereas no such deviation from 1:1 was observed in melanistic snakes (chi(Andren and Nilson 1981)=1.8; *p* = 0.18; Fig. 2).

Our literature review unearthed reports on just eight terrestrial species where melanism was studied in relation to size-related variables, sex ratio, or sex-specific prevalence. These studies included four oviparous species (*Natrix natrix*, *Hierophis viridiflavus*, *Heterodon platirhinos*, and *Elaphe quadrivirgata*), as well as four viviparous species (*Vipera berus*, *V. aspis*, *Vipera renardi*, and *Thamnophis sirtalis*). For other species, no further data were available, despite melanism being reported. For example, the occurrence of melanism in dice snakes (*Natrix tessellata*) was frequently described, but we failed to find any report of its effects on body size or sex ratio (Mebert 2011; Ajtić et al. 2013). Given the limited number of species for which we found data on the effects of melanism, we could not perform formal statistical tests and our analysis is limited to descriptive statistics. The studies we have summarized show that melanism in viviparous species tends to have a positive impact on body size or related variables (8/17 studies report a positive effect, 8/17 report no effect, and 1/17 report a negative effect) in both males (3 reports) and females (4 reports) or without sex distinction (3 reports). Moreover, in most cases, melanism in viviparous species is more common in females than in males (7/15 studies report a female bias, 3/15 report a male bias, and 5/15 report no effect). Less clear is the picture for oviparous snakes. Excluding our study, 2/5 studies reported a negative effect of melanism on size-related traits, 2/5 reported a positive effect, and 1/5 reported no effect. The prevalence of melanism does not seem to show a clear pattern either, with 2/6 studies indicating a higher frequency of melanism in males, 1/6 in females, and 3/6 with no tendency. The addition of our study, however, tilts the balance towards a negative effect of melanism on body size (3/6 reports of a negative effect of melanism on size and 3/7 reports indicating a tendency towards increased prevalence in males rather than females).

## Discussion

Our study is one of the few to clearly show a negative association between melanistic phenotype and body size in an oviparous species, the European grass snake. We have ruled out a potential bias towards capturing individuals of a given size, because the snakes were captured randomly in a wide spectrum of habitats, throughout the season, though mostly in the post-mating period, and juveniles were not incorporated in the analysis. The documented pattern stands contrary to our predictions and the generally reported or anticipated patterns (e.g., Andren and Nilson 1981; Madsen and Stille 1988; Forsman 1995a). The magnitude of size differences between phenotypes remains similar in both sexes, whereas sex-specific effects of the black phenotype appear to occur at the sex ratio level. In the typical phenotype, we observed a clear female-biased sex ratio, in line with other data from southern Poland (Juszczyk 1987), but contrary to that generally expected for species with a genetically determined sex ratio of 1:1 (Fisher 1999; Shine and Bull 1977). Such female bias could be the outcome of a high local productivity possibly underlying a high abundance of reptiles in general (Błażuk 2007) and be beneficial for females, the sex that bears much higher costs of reproduction. In the melanistic fraction, however, we did not observe such a pattern, as the sex ratio did not differ from 1:1, which may be due to the smaller proportion of females and/or the greater proportion of males compared with the typical phenotype. Although the effects of the melanistic phenotype on both sexes seem to differ between the individual and the population levels, we consider the responses on both scales to be complementary and driven by common environmental factors.

The smaller sizes of melanistic snakes may represent an outcome of at least two non-mutually exclusive factors: the pleiotropic effect of black phenotype expression (Ducrest et al. 2008) and the high removal rate of larger melanistic individuals by predators. Whereas we cannot exclude the former factor as being responsible for our results, the latter one is partially corroborated by the pattern observed at the sex ratio level. In the grass snake, females are well known for achieving larger sizes, a pattern confirmed in this study, and known to promote predation risk (Wellborn 1994; Niskanen and Mappes 2005). Such size bias, combined with the impaired antipredatory function of the black phenotype (Madsen 1987), may impose stronger predation pressure on females. This, in turn, may lead to a reduced proportion of females in the melanistic population fraction compared with the typical one.

A smaller size is generally assumed to have a negative impact on reptiles’ survival (Civantos et al. 1999) and fecundity (Olsson 1993). Therefore, the persistence of the melanistic phenotype in the studied population (Błażuk 2007), despite its negative effect on body size, suggests that black coloration must have beneficial consequences for other features. Anti-UV protection driven by high-melanin concentration seems to be of minor importance because the keratinized outermost layer is itself a good anti-UV barrier (Tercafs 1963; Chang and Zheng 2003). Also unlikely is the role of melanism in mate selection, since snakes do not appear to rely on visual cues (Andren and Nilson 1981). Melanism expression is, however, suggested as improving defense against pathogens (Roulin 2014). This effect has already been demonstrated in endotherms, but in ectotherms, the elevated immune response has only recently been demonstrated in melanistic lizards (Vroonen et al. 2013; Seddon and Hews 2016; Baeckens and Van Damme 2018). The positive impact of melanism on immunity could be more pronounced in males, which are generally known to exhibit a lower immune response compared with females (Saad and Shoukrey 1988). Such an asymmetric effect in favor of males could also explain the higher proportion of this sex in the melanistic phenotype compared with the typical one.

Our systematic review of the available data points to an emerging pattern of the negative impact of the melanistic phenotype on body size in oviparous snakes (Table 2). Conversely, in viviparous species, there is a discernible tendency towards a positive effect of melanism on size-related traits and a clear female-biased prevalence of the melanistic phenotype. Such a discrepancy between reproductive modes can be attributed to the different costs of reproduction borne by females. In general, one can expect the energetic costs of reproduction for females to be lower in oviparous species than in viviparous species, owing to the shorter time of embryo retention in the body cavity and the longer foraging time window before the onset of the winter (Madsen and Shine 1992; Gregory et al. 1999). This may weaken the impact of thermal benefits and lead to the suggested reduction in larger individuals and females as a consequence of predation (Andren and Nilson 1981; Madsen 1987), indicated not only here in grass snakes, but in other oviparous species as well. The universality of this pattern requires studies of other species, preferably in conjunction with data on individuals’ ages, in order to rule out the possibility of the negative effect of melanism on growth/body size instead of large-specimen removal. Such an anticipated specificity of melanism effects relative to reproductive mode may further drive the diversity of population responses towards environmental change. Future studies on the consequences of melanism should include a wider array of features in a sex-specific context if we are to broaden our understanding of the mechanisms underlying the persistence of polymorphism beyond thermal benefits. Such data could hint at a link between melanism prevalence and population viability, an aspect especially relevant in the light of climate change.

**Table 2:** Association between the melanism, body size (SVL – snout-vent length), and sex-specific melanism frequency or sex ratio in terrestrial snakes reported in published studies

Species	Region	Body size, mass, or condition	Sex-specific frequency or sex ratio	Reproductive mode	Source
*Natrix natrix*	Bieszczady Mts., Poland	Reduced SVL in melanistic males and females	1:1 sex ratio in melanistic with female-bias in typically colored snakes	Oviparous	This study
*Natrix natrix*	Gotland, Sweden	No data	Male-biased sex ratio in melanistic snakes	Oviparous	Nilson and Andren (1981)
*Natrix natrix*	Cyprus	Reduced body mass in melanistic females No effect in males	Higher frequency in females	Oviparous	Blosat (1997)
*Hierophis viridiflavus*	Sagittario Valley, Italy	Increased SVL, mass, and condition in melanistic males and females	Similar frequency in both sexes	Oviparous	Luiselli (1995)
*Hierophis viridiflavus*	Various sites, Italy	Reduced SVL in melanistic males and females	Higher frequency in males	Oviparous	Zuffi (2008)
*Heterodon platirhinos*	Various sites, USA	Increased SVL in melanistic males and females	Similar frequency in both sexes	Oviparous	Edgren (1957)
*Elaphe quadrivirgata*	Yakushima Island, Japan	No effect on SVL, mass, or condition in males and females	Similar frequency in both sexes	Oviparous	Tanaka (2009)
*Thamnophis sirtalis*	Lake Erie, Canada	Increased SVL in melanistic males and females	Similar frequency in both sexes	Viviparous	Gibson and Falls (1988)
*Thamnophis sirtalis*	Lake Erie, Canada	No effect on SVL In males and females	Similar frequency in both sexes	Viviparous	King (1988)
*Vipera renardi*	Krasnodar Region, Russia	No effect on SVL and mass in males and females	Similar frequency in both sexes	Viviparous	Ostrovskikh (1997)
*Vipera aspis*	The Alps, Switzerland	Reduced SVL and mass in melanistic males and females Faster growth in melanistic snakes	Higher frequency in females	Viviparous	Monney et al. (1996)
*Vipera aspis*	The Alps, Switzerland	Enhanced condition in melanistic females in 2 out of 4 sites No effect on SVL	Similar frequency in both sexes	Viviparous	Castella et al. (2013)
*Vipera berus*	Hallands Vadero, Sweden	Greater mass, but not SVL in melanistic males and females	Higher frequency in females	Viviparous	Andren and Nilson (1981)
*Vipera berus*	The Alps, Italy	Greater SVL in melanistic females No data on males	Higher frequency in females	Viviparous	Luiselli (1992)
*Vipera berus*	The Alps, Italy	Greater mass and better condition in melanistic males No data on females	Higher frequency in females	Viviparous	Luiselli (1993)
*Vipera berus*	The Alps, Italy	No data	Higher frequency in females	Viviparous	Luiselli et al. (1994)
*Vipera berus*	The Alps, Switzerland	Greater SVL and mass in melanistic males and females	Higher frequency in females	Viviparous	Monney et al. (1995)
*Vipera berus*	The Alps, Switzerland	Greater SVL and mass in melanistic males and females Faster growth in melanistic snakes	Higher frequency in females	Viviparous	Monney et al. (1996)
*Vipera berus*	Uppsala and islands, Sweden	No effect on SVL in males and females	No data	Viviparous	Forsman (1991)
*Vipera berus*	Uppsala, coast and islands, Sweden	No effect on SVL in males and females	Higher frequency in males at 2 out of 9 sites		Forsman (1995a)
*Vipera berus*	Angskar islands, Sweden	No effect on growth in males and females	No data	Viviparous	Forsman (1993)
*Vipera berus*	Angskar islands, Sweden	No effect on SVL, mass, and condition in males No data on females	Similar frequency in both sexes	Viviparous	Forsman and Ås (1987)
*Vipera berus*	Hallands Vadero, Sweden	Greater size, mass, and faster growth in melanistic snakes No sex distinction	No data	Viviparous	Madsen and Stille (1988)
*Vipera berus*	The Carpathians, Romania	No effect on SVL in males and females	Higher frequency in males	Viviparous	Strugariu and Zamfirescu (2011)
*Vipera berus*	Hungary	Increased SVL and mass in melanistic snakes. No sex distinction	Higher frequency in males	Viviparous	Újvári et al. 2001

## Data Availability

Data are accessible here: 10.6084/m9.figshare.8971118.v1

## References

[CR1] Ajtić R, Tomović L, Sterijovski B, Crnobrnja-Isailović J, Djordjević S, Djurakić M, Golubovic A, Simovic A, Arsovski D, Andjelkovic M, Krstić M, Sukalo G, Gvozdenovic S, Aidam A, Louise Michel C, Ballouard J-M, Bonnett X (2013). Unexpected life history traits in a very dense population of dice snakes. Zool Anz.

[CR2] Andren C, Nilson G (1981). Reproductive success and risk of predation in normal and melanistic colour morphs of the adder, *Vipera berus*. Biol J Linn Soc.

[CR3] Baeckens S, Van Damme R (2018). Immunocompetence and parasite infestation in a melanistic and normally-coloured population of the lacertid lizard, *Podarcis siculus*. Amphibia-Reptilia.

[CR4] Błażuk J (2007). Herpetofauna doliny Sanu pod Otrytem i terenów przyległych (Bieszczady Zachodnie). Gady. Roczniki Bieszczadzkie.

[CR5] Blosat BA, Böhme W (1997) Melanism in a population of *Natrix natrix* in Cyprus. In: Rocek ZS Hart (eds.). Abstracts of the Third World Congress of Herpetology, Prag: 22

[CR6] Böhme W, Wiedl H (1994). Status and zoogeography of the herpetofauna of Cyprus, with taxonomic and natural history notes on selected species (genera Rana, Coluber, Natrix, Vipera). Zool Middle East.

[CR7] Borczyk B (2004). *Natrix natrix* (European grass snake) male combat. Herp Rev.

[CR8] Borczyk B (2007). The causes of intraspecific variation in sexual dimorphism in the common grass snake populations, *Natrix natrix* Linnaeus, 1758 (Serpentes, Colubridae): data from the South Western Poland. Acta Zool Cracov A.

[CR9] Bury S, Zając B (2020) The loss of sexual size dimorphism in urban populations of a widespread reptile, the European grass snake *Natrix natrix*. Curr Zool zoz034. 10.1093/cz/zoz034 10.1093/cz/zoz034 PMC7233604 32440281

[CR10] Castella B, Golay J, Monney JC, Golay P, Mebert K, Dubey S (2013). Melanism, body condition and elevational distribution in the asp viper. J Zool.

[CR11] Chang C, Zheng R (2003). Effects of ultraviolet B on epidermal morphology, shedding, lipid peroxide and antioxidant enzymes in Cope’s rat snake (*Elaphe taeniura*). J Photochem Photobiol B.

[CR12] Civantos E, Salvador A, Veiga JP (1999). Body size and microhabitat affect winter survival of hatchling *Psammodromus algirus* lizards. Copeia.

[CR13] Dubey S, Roulin A (2014). Evolutionary and biomedical consequences of internal melanins. Pigment Cell Melanoma Res.

[CR14] Ducrest AL, Keller L, Roulin A (2008). Pleiotropy in the melanocortin system, coloration and behavioural syndromes. Trends Ecol Evol.

[CR15] Edgren RA (1957). Melanism in hog-nosed snakes. Herpetologica.

[CR16] Fisher RA (1999). The genetical theory of natural selection: a complete.

[CR17] Forsman A (1991). Variation in sexual size dimorphism and maximum body size among adder populations: effects of prey size. J Anim Ecol.

[CR18] Forsman A (1993). Growth rate in different colour morphs of the adder, *Vipera berus*, in relation to yearly weather variation. Oikos.

[CR19] Forsman A (1995). Opposing fitness consequences of colour pattern in male and female snakes. J Evol Biol.

[CR20] Forsman A (1995). Heating rates and body temperature variation in melanistic and zigzag *Vipera berus*: does colour make a difference?. Ann Zool Fenn.

[CR21] Forsman A, Ås S (1987). Maintenance of colour polymorphism in adder, *Vipera berus*, populations: a test of a popular hypothesis. Oikos.

[CR22] Forsman A, Ahnesjö J, Caesar S, Karlsson M (2008). A model of ecological and evolutionary consequences of color polymorphism. Ecology.

[CR23] Gibson AR, Falls JB, Downhower JF (1988). Melanism in the common garter snake: a Lake Erie phenomenon. The Biogeography of the Island Region of Western Lake Erie.

[CR24] Gregory PT, Crampton LH, Skebo KM (1999). Conflicts and interactions among reproduction, thermoregulation and feeding in viviparous reptiles: are gravid snakes anorexic?. J Zool.

[CR25] Juszczyk W (1987) Plazy i gady krajowe. Państwowe Wydawnictwo Naukowe

[CR26] Kemp DJ, Rutowski RL, Mendoza M (2005). Colour pattern evolution in butterflies: a phylogenetic analysis of structural ultraviolet and melanic markings in North American sulphurs. Evol Ecol Res.

[CR27] King RB (1988). Polymorphic populations of the garter snake *Thamnophis sirtalis* near Lake Erie. Herpetologica.

[CR28] Luiselli L (1992). Reproductive success in melanistic adders: a new hypothesis and some considerations on Andrén and Nilson’s (1981) suggestions. Oikos.

[CR29] Luiselli L (1993) The ecological role of color polymorphism in male adders, *Vipera berus*: testing the hypotheses. Rev Ecol 48:49–56

[CR30] Luiselli L (1995) Body size, sexual size dimorphism and reproduction in different colour morphs in a population of western whip snakes, *Coluber viridiflavus*. Rev Ecol 50:365–376

[CR31] Luiselli L, Capula M, Rugiero L, Anibaldi C (1994). Habitat choice by melanistic and cryptically coloured morphs of the adder, *Vipera berus*. Ital J Zool.

[CR32] Madsen T (1987). Are juvenile grass snakes, *Natrix natrix*, aposematically coloured?. Oikos.

[CR33] Madsen T, Shine R (1992). Determinants of reproductive success in female adders, *Vipera berus*. Oecologia.

[CR34] Madsen T, Shine R (1993). Costs of reproduction in a population of European adders. Oecologia.

[CR35] Madsen T, Stille B (1988). The effect of size dependent mortality on colour morphs in male adders, *Vipera berus*. Oikos.

[CR36] Mebert K (ed.) (2011) The dice snake, *Natrix tessellata*: biology, distribution and conservation of a Palaearctic species. DGHT, Germany

[CR37] Monney JC, Luiselli L, Capula M (1995). Correlates of melanism in a population of adders (*Vipera berus*) from the Swiss Alps and comparisons with other alpine populations. Amphibia–Reptilia.

[CR38] Monney JC, Luiselli L, Capula M (1996). Body size and melanism in *Vipera aspis* in the Swiss Prealps and central Italy and comparison with different Alpine populations of *Vipera berus*. Rev Suisse Zool.

[CR39] Nilson G, Andren C (1981). Morphology and taxonomic status of the grass snake, *Natrix natrix* (L.) (Reptilia, Squamata, Colubridae) on the island of Gotland, Sweden. Zool J Linnean Soc.

[CR40] Niskanen M, Mappes J (2005). Significance of the dorsal zigzag pattern of Vipera latastei gaditana against avian predators. J Anim Ecol.

[CR41] Olsson M (1993). Male preference for large females and assortative mating for body size in the sand lizard (*Lacerta agilis*). Behav Ecol Sociobiol.

[CR42] Ostrovskikh SV (1997). Different forms of melanism and its development with age in the populations of steppe viper *Vipera renardi* (Christoph, 1861). Russ J Herpetol.

[CR43] Roulin A (2014). Melanin-based colour polymorphism responding to climate change. Glob Chang Biol.

[CR44] Roulin A, Riols C, Dijkstra C, Ducrest AL (2001). Female plumage spottiness and parasite resistance in the barn owl (*Tyto alba*). Behav Ecol.

[CR45] Saad AH, Shoukrey N (1988). Sexual dimorphism on the immune responses of the snake, Psammophis sibilans. Immunobiology.

[CR46] Seddon RJ, Hews DK (2016). Phenotypic correlates of melanization in two *Sceloporus occidentalis* (Phrynosomatidae) populations: behavior, androgens, stress reactivity, and ectoparasites. Physiol Behav.

[CR47] Shine R, Bull JJ (1977). Skewed sex ratios in snakes. Copeia.

[CR48] Strugariu A, Zamfirescu ŞR (2011). Population characteristics of the adder (*Vipera berus berus*) in the Northern Romanian Carpathians with emphasis on colour polymorphism: is melanism always adaptive in vipers?. Anim Biol.

[CR49] Tanaka K (2009). Does the thermal advantage of melanism produce size differences in color-dimorphic snakes?. Zool Sci.

[CR50] Tercafs R (1963). Transmission of ultra-violet, visible and infra-red radiation through the keratinous layer of reptile skin (Serpentes and Sauria). Ecology.

[CR51] Trullas SC, van Wyk JH, Spotila JR (2007). Thermal melanism in ectotherms. J Therm Biol.

[CR52] Újvári B, Lazányi I, Farkas B, Korsós Z (2001). An isolated adder (*Vipera berus*) population in Hungary.

[CR53] Vroonen J, Vervust B, Van Damme R (2013). Melanin based colouration as a potential indicator of male quality in the lizard *Zootoca vivipara* (Squamata: Lacertidae). Amphibia-Reptilia.

[CR54] Wellborn GA (1994). Size-biased predation and prey life histories: a comparative study of freshwater amphipod populations. Ecology.

[CR55] Zuffi M (2008). Colour pattern variation in populations of the European Whip snake, Hierophis viridiflavus: does geography explain everything?. Amphibia-Reptilia.

